# Heterogeneity in Cation Exchange Ag^+^ Doping of CdSe Nanocrystals

**DOI:** 10.1021/acsnanoscienceau.3c00010

**Published:** 2023-04-25

**Authors:** Abigail Freyer, Trevor M. Tumiel, Michelle Smeaton, Benjamin H. Savitzky, Lena F. Kourkoutis, Todd D. Krauss

**Affiliations:** ∇ Department of Chemistry, University of Rochester, Rochester, New York 14627-0216, United States; ‡ The Institute of Optics, University of Rochester, Rochester, New York 14627-0216, United States; § Department of Materials Science and Engineering, Cornell University, Ithaca, New York 14853, United States; ∥ Department of Physics, Cornell University, Ithaca, New York 14853, United States; ⊥ School of Applied and Engineering Physics, Cornell University, Ithaca, New York 14853, United States; # Kavli Institute at Cornell for Nanoscale Science, Cornell University, Ithaca, New York 14853, United States

**Keywords:** cation exchange, semiconductor nanocrystals, atomic force microscopy, scanning transmission electron microscopy electron energy loss spectroscopy, single molecule spectroscopy

## Abstract

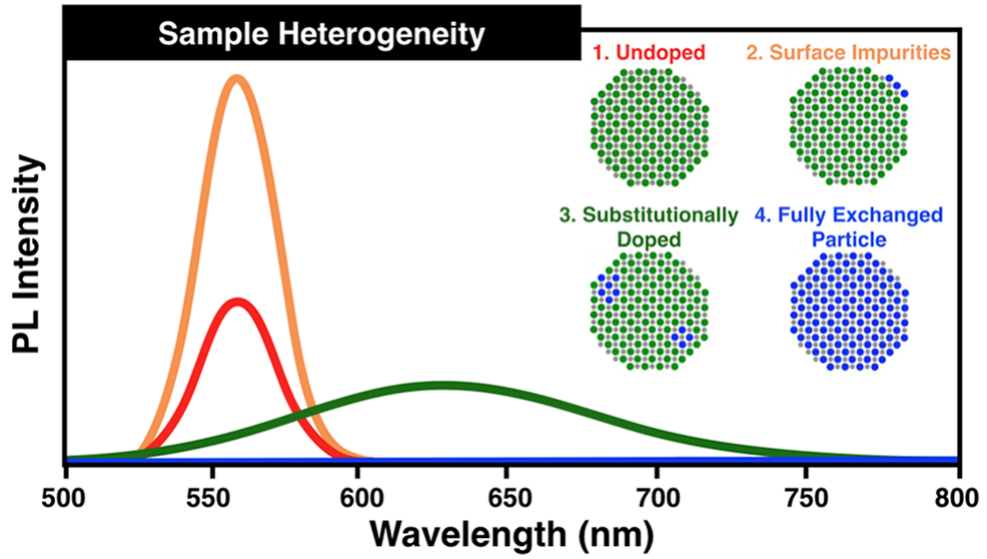

Cation exchange is becoming extensively used for nanocrystal (NC) doping in order to produce NCs with unique optical and electronic properties. However, despite its ever-increasing use, the relationships between the cation exchange process, its doped NC products, and the resulting NC photophysics are not well characterized. For example, similar doping procedures on NCs with the same chemical compositions have resulted in quite different photophysics. Through a detailed single molecule investigation of a postsynthesis Ag^+^ doping of CdSe NCs, a number of species were identified within a single doped NC sample, suggesting the differences in the optical properties of the various synthesis methods are due to the varied contributions of each species. Electrostatic force microscopy (EFM), electron energy loss spectroscopy (EELS) mapping, and single molecule photoluminescence (PL) studies were used to identify four possible species resulting from the Ag^+^-CdSe cation exchange doping process. The heterogeneity of these samples shows the difficulty in controlling a postsynthesis cation exchange method to produce homogeneous samples needed for use in any potential application. Additionally, the heterogeneity in the doped samples demonstrates that significant care must be taken in describing the ensemble or average characteristics of the sample.

Just as with bulk crystalline material, semiconductor nanocrystals (NCs) can be doped with impurity atoms in order to control different material properties that tailor the semiconductor for electronic, optical, and magnetic applications.^1−4^ Specifically, aliovalent doping can introduce free charge carriers through extra electrons or holes offered by the dopant, with the resulting doped material quite useful for electronic applications. In particular, doping NCs aliovalently have been shown to create interesting optical and charge characteristics,^3,5−7^ including a charge dependent photoluminescence (PL) intensity.^5^

A common technique for doping is cation exchange,^8−16^ including postsynthesis^9,17^ and one-pot^6,18^ cation exchanges, especially for the Ag^+^-CdSe NC exchange.^3,5,6^ Cation exchange is an extremely important synthesis technique not only to produce doped NCs but also to facilitate the conversion of one NC to a chemically different NC, assembling structures that cannot be produced directly.^19−22^ The benefits of the postsynthesis cation exchange for doping of NCs include increased control over the doping level and the availability of an undoped sample for direct comparison to the doped samples,^3^ while the one-pot exchange allows the dopant to be introduced during or immediately following the NC synthesis.^6,18^

While it is clear that doping induces changes in the optical properties of NCs, different configurations of charged impurities cause variations in the resulting NC photophysics.^3,5−7,23,24^ For example, for Ag^+^ doped CdSe NCs, the postsynthesis exchange resulted in an enhanced exciton PL intensity and growth of a weak dopant peak at lower energies relative to the band edge fluorescence.^3,5^ Conversely, in situ exchange has been shown to quench the exciton peak while producing intense, broad, red-shifted dopant PL.^6^ Also, it was recently shown that the PL response from Ag^+^ doped CdSe NCs is highly sensitive to the exact placement of the impurity atoms around the NC.^5^ With both doping techniques, the absorbance spectrum did not have an observable change with the addition of Ag^+^, which is unexpected for a NC sample that has increasing amounts of Ag^+^ dopant added, as a measurable amount of Ag_2_Se should eventually be produced by the cation exchange. Though the doping method varied, in principle the resulting Ag^+^ doped CdSe NCs should be similar in chemical composition and, thus, should possess similar photoluminescent properties. For example, one may expect to first order that the NCs would contain a Poisson distribution of impurity atoms, implying similar photophysics for each NC. These discrepancies suggest that a homogeneously doped NC product may be a poor assumption, and thus ensemble optical spectroscopic methods are likely insufficient to fully understand the doped NC photophysics. Rather, single molecule methods must be utilized to tease out the heterogeneous nature of the doped NCs.

Here we present single molecule spectroscopic and force microscopic studies of individual CdSe NCs aliovalently doped with Ag^+^ produced via the postsynthesis process. Scanning transmission electron microscopy electron energy loss spectroscopy (STEM-EELS) maps revealed both heavily doped and fully exchanged particles, as well as undoped NCs, within a single sample. A distribution of nanocrystal charge magnitudes was observed within a single doped sample using electrostatic force microscopy (EFM), suggesting that each doped NC has differing numbers of incorporated impurities despite undergoing the same doping conditions. Lastly, single molecule PL measurements revealed that individual NCs exhibit either exciton or dopant PL, but never both. Altogether, we conclude that the cation exchange doping process ultimately creates a variety of species, including undoped NCs, lightly doped NCs, NCs with impurity ions sitting on the surface, and fully exchanged NCs. These results imply that a postsynthesis Ag^+^ cation exchange doping of CdSe is difficult to control with the current procedures, which could present a significant challenge when the desired applications require NCs of uniform quality and composition.

Ag^+^ doped CdSe NCs were synthesized^25,26^ and impurity doped^3^ via a postsynthesis cation exchange as reported previously.^5^ Since the amount of dopant actually incorporated into the NCs is typically far less than what is added,^5^ the dopant concentration was quantified using ICP-MS as detailed in the Supporting Information (SI). Based on the ICP-MS data (Figure S1) the average Ag per NC was quite high, suggesting very heavily doped particles. Indeed, for the largest amounts of Ag^+^ added, measured dopant concentrations neared those expected for fully exchanged particles (i.e., hundreds of Ag^+^ per NC). However, linear optical spectroscopic measurements, although agreeing with previous reports,^3,5^ actually suggested the opposite. For example, minimal changes in absorbance spectra were observed across a wide range of dopant concentrations, even when high doping levels were achieved (Figure 1a). Additionally, ensemble PL spectra were dominated by a peak from the band edge exciton, with only a relatively minor dopant peak to the red of the main peak, the latter of which increases in intensity with increasing dopant concentration (Figure 1b).

**Figure 1:**
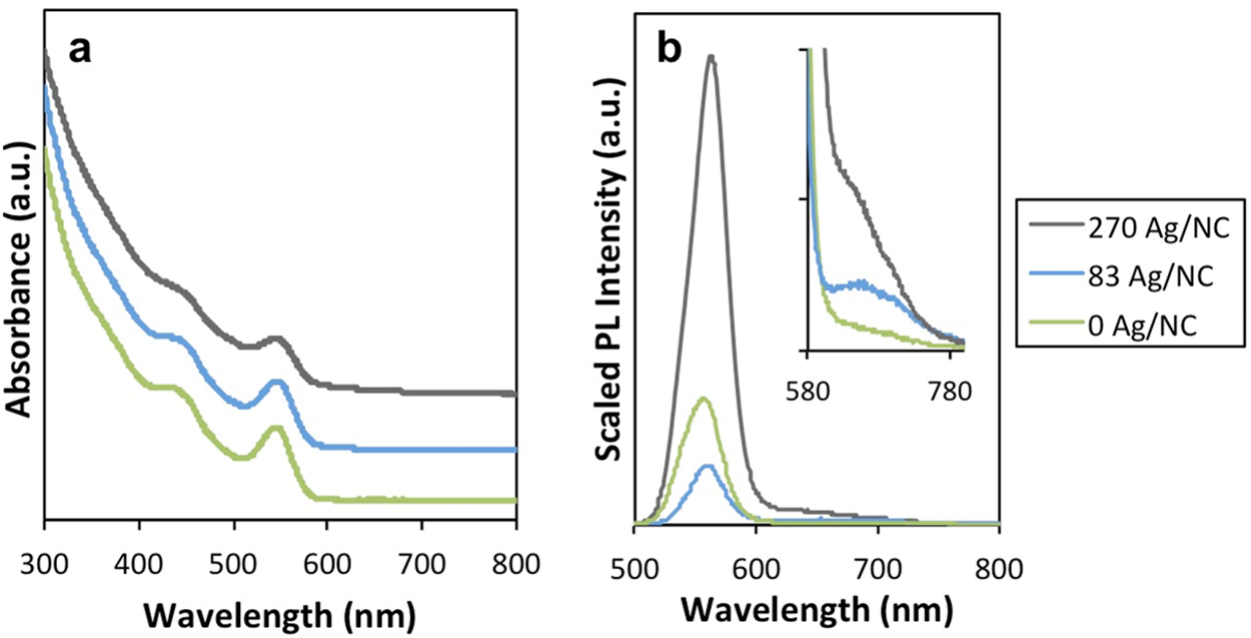
Ensemble (a) absorbance and (b) PL spectra for two Ag^+^ doped CdSe samples and the corresponding undoped CdSe sample for a single series of doping. The inset in (b) magnifies the weak PL feature near 700 nm.

Using EFM (see SI for EFM procedures), the charges of dozens of individual NCs were measured across a number of samples with varying Ag^+^ concentrations. Sample EFM images for a NC doping series are displayed in Figure S4 showing the charge on each NC. From these images, a histogram of measured charges per NC can be determined, as shown in Figure 2. As the Ag^+^ concentration per NC was varied, we found the median charge per NC and the shape and width of the distributions varied. For example, an undoped NC sample yielded a symmetric distribution with an average neutral charge value (Figure 2a), as expected for nominally neutral NCs. Note that the undoped sample was subject to the same procedure as the doped samples, just without the added Ag salts, including additional washings and exposure to ethanol. Thus, the slight charge on the order of 1*e* observed for some members of this undoped sample is attributed to slight differences in ligand coverage (i.e., exposed Cd^2+^ or Se^2–^ surface atoms) present around each NC as a result of this procedure.^5^ For a doped sample with 83 Ag/NC, the distribution still exhibited an average neutral charge but was skewed asymmetrically toward more positive charges (Figure 2b). As the Ag/NC dopant level increased further, the distribution became broader and a larger fraction of the NCs demonstrated a more positive charge (Figure 2c). Altogether, the broadening in the charge histograms suggests that as more Ag^+^ dopant is added, the NCs within a given sample become more heterogeneous with respect to numbers of Ag^+^ per NC.

**Figure 2:**
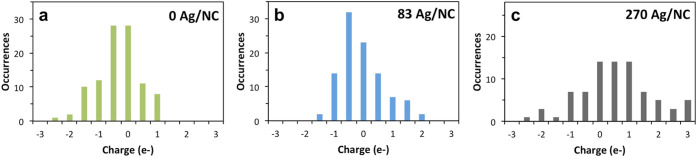
Charge histograms for a set of Ag^+^ doped CdSe samples. (a) 0 Ag/NC, (b) 83 Ag/NC, (c) 270 Ag/NC.

To better understand how Ag^+^ is incorporated into the CdSe NCs during the cation exchange doping process, STEM-EELS was used to locate the Ag relative to the Cd in the NC samples. On a particle-to-particle basis, there were significant differences in the amount and location of Ag. Representative data for what was observed is illustrated for the doped sample with 83 Ag/NC in Figure 3. For some NCs, the Ag dopant is localized around, either on or near, the nanoparticle (Figure 3a,b). However, there were also some larger, less uniform particles present with much greater amounts of Ag, which we assume to be fully exchanged Ag_2_Se particles (Figures 3c–e). No matter the doping level, large clusters of Ag_2_Se were always observed and became more prevalent as dopant level increased. Thus, the STEM-EELS strongly suggests that doping produces a range of heterogeneously exchanged particles, from some NCs that appear to be lightly doped, to large particles that appear to be fully exchanged Ag_2_Se. To further uncover the suspected heterogeneity of the doped NC samples, we performed single molecule PL spectroscopy experiments to investigate the PL character of individual NCs. For the undoped sample, exciton PL was difficult to measure on the single particle level, due to rapid PL quenching, as demonstrated by the large signal-to-noise in the PL spectra presented in Figure 4a. For the medium doped 83 Ag/NC sample, the majority of the NCs displayed dopant PL features (Figure 4b); however, exciton PL was still observed for a number of individual NCs. For the more heavily doped 270 Ag/NC sample, both exciton and dopant PL was observed similar to the 83 Ag/NC sample, but the PL features were routinely more intense (Figure 4c). In all samples studied, individual particles demonstrated either exciton PL or dopant PL, never both. This intriguing result suggests that there were at least two different species of emissive NCs present within a doped sample: one that facilitated exciton PL emission and one that facilitated dopant PL emission.

**Figure 3:**
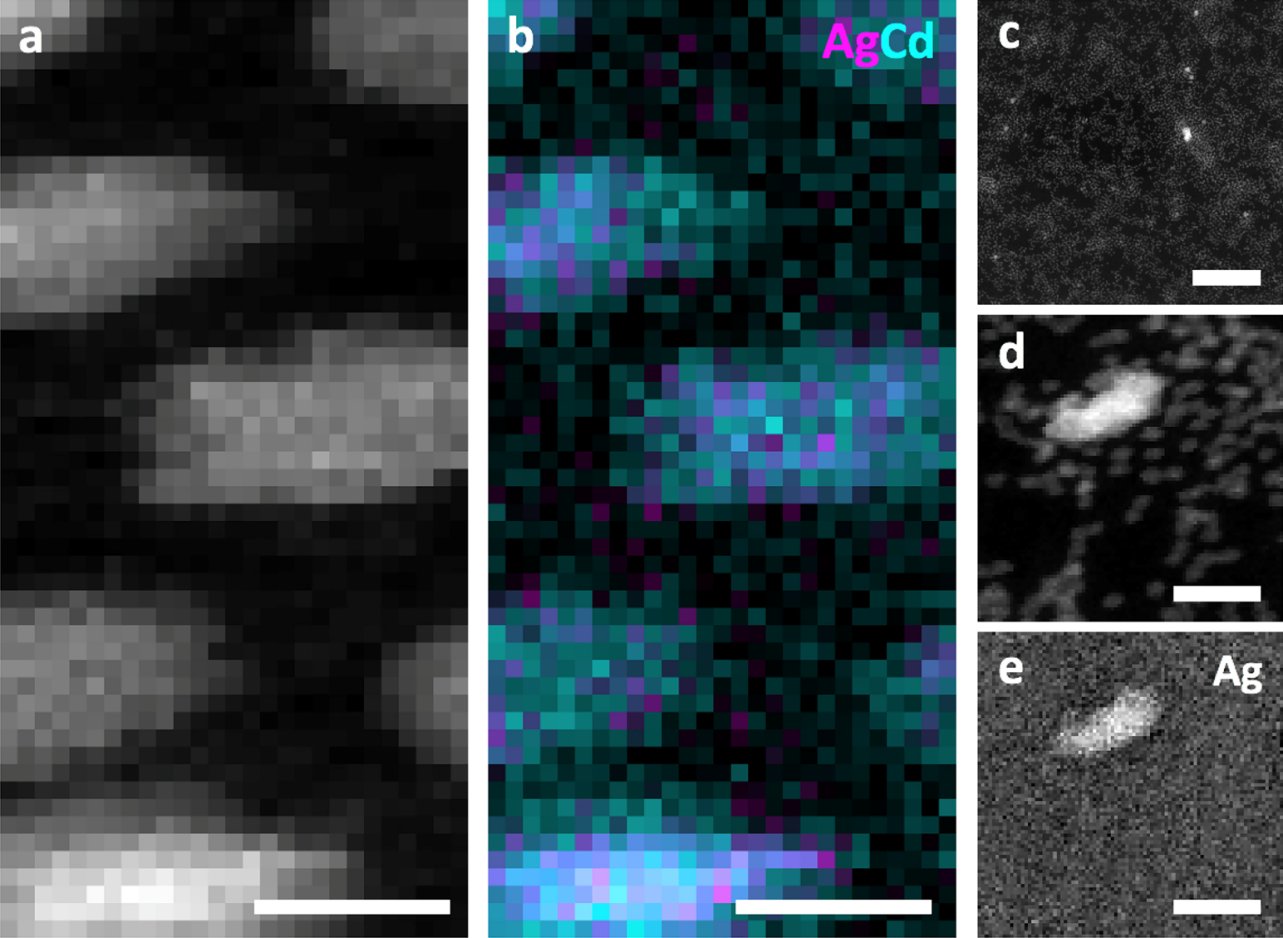
(a) Simultaneously acquired ADF-STEM image. (b) Corresponding false-colored Ag (magenta) and Cd (cyan) EELS elemental maps for the 83 Ag/NC Ag^+^ doped CdSe sample. (c,d) Lower magnification ADF-STEM images of the 83 Ag/NC Ag^+^ doped CdSe sample and (e) Ag EELS elemental map acquired simultaneously with (d). Scale bars are 2 nm in (a,b), 100 nm in (c), and 20 nm in (d,e). Note that the NCs have an oblong shape in the STEM-EELS images due to thermal stage drift during the EELS measurement.

**Figure 4:**
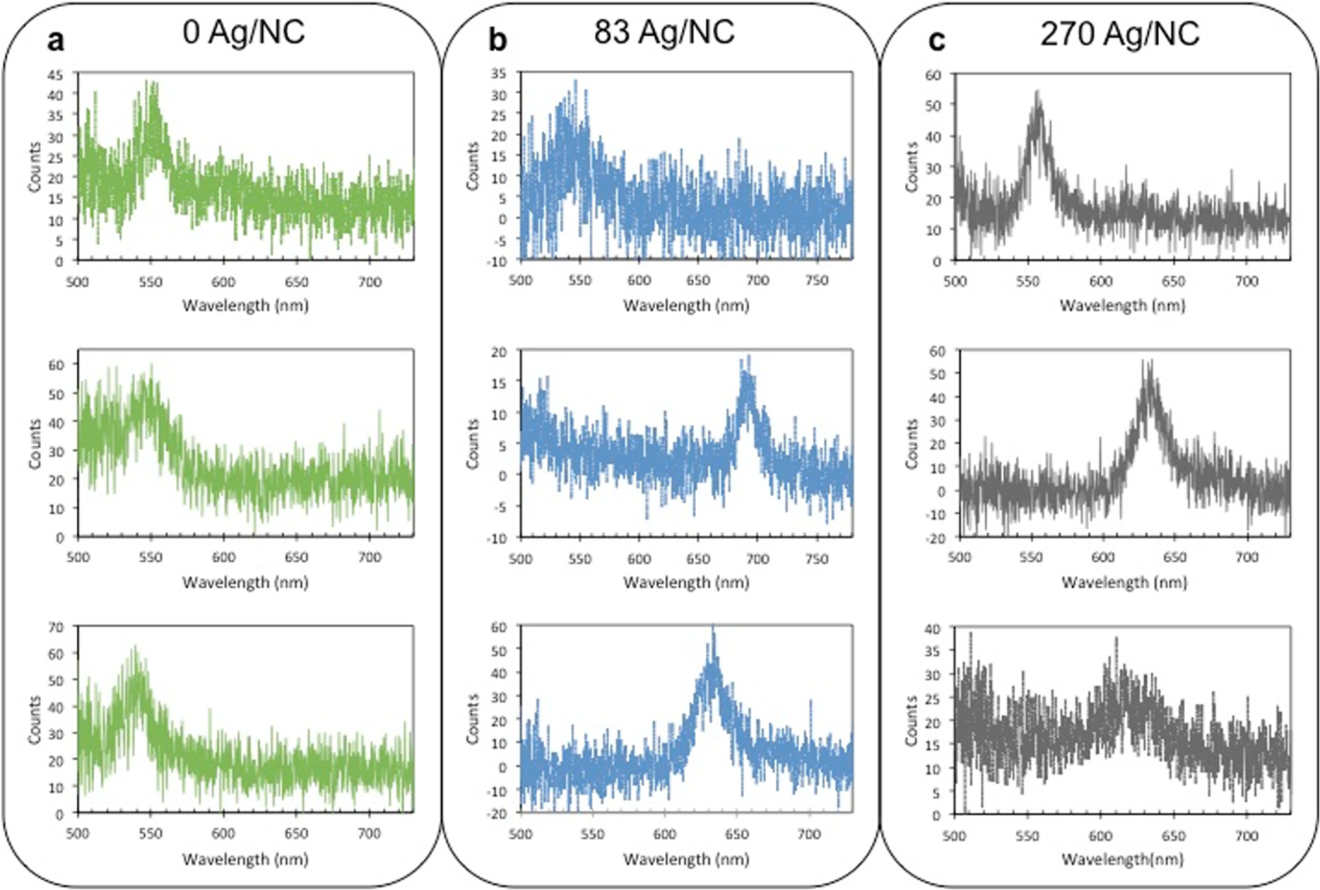
Representative single molecule PL spectra for the samples of (a) 0 Ag/NC, (b) 83 Ag/NC, and (c) 270 Ag/NC showing the variation in PL signatures found within each sample.

Single molecule PL spectroscopy has as an inherent limitation stipulating that the only nanoparticles observed are those that are highly fluorescent. To address this limitation, we performed correlated atomic force microscopy (AFM) and single molecule PL measurements for the same area (Figure S5). These measurements revealed that the magnitude of nanoparticles actually present was on the order of 50 times larger than that of the bright NCs readily observed via single molecule PL. The large fraction of dark NCs arises partially due to efficient PL quenching or bleaching of undoped or lightly doped CdSe NCs. Importantly, the lack of any measurable PL spectra can also be attributed to any NCs that have fully converted to Ag_2_Se, thus supporting the hypothesis that the doped sample is highly heterogeneous.

Overall, the combined EFM, STEM-EELS, and single molecule PL spectroscopy suggest that the cation exchange process which yields Ag^+^ doped CdSe NCs produces four possible species of particles that can be present in a single sample (Figure 5). First, there may be some native CdSe NCs left undoped, which would present a neutral charge, display no Ag in or around the NCs, and display exciton PL emission. In the second species type, some Ag^+^ impurity ions sit near the surface of the NC or find their way into an interstitial site, resulting in a particle that exhibits a measurable positive charge. For this species type, some Ag would be present in or near the NCs and more intense exciton PL would be observed due to the symmetry breaking of the NCs by the charged impurity, causing the brightening of a dark electronic state of the NC.^5,27,28^ For NCs that become doped through substitutional doping with exchange of some Cd^2+^ ions with Ag^+^ dopants, the NC would largely remain neutral as two Ag^+^ dopants replace a single Cd^2+^ host ion. For these NCs, only the dopant PL emission would be detected by single molecule PL measurements.

**Figure 5:**
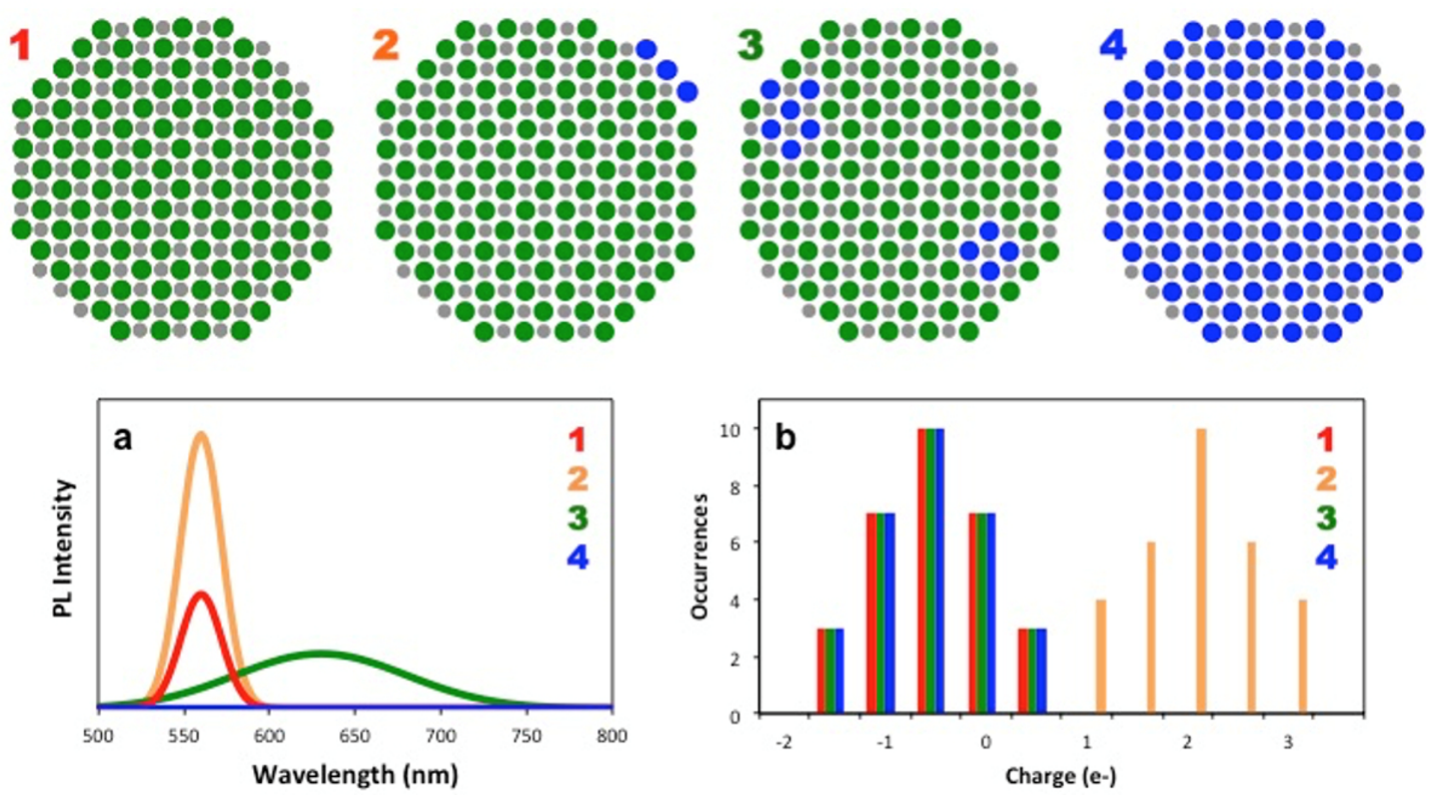
(top) Schematic of four possible product species in the Ag^+^ doped CdSe samples. Green circles represent Cd atoms, gray represents Se atoms, and blue represents Ag atoms. The colored numbers associated with each species refer to the colors in the plots in (a) and (b). (bottom) Illustrative representation of how each species 1–4 could contribute to the (a) ensemble level PL spectrum and (b) the average charge distribution for a Ag+ doped CdSe sample. Note that a combination of the schemes may be possible, which could lead to a unique combination of PL emission and measured charge that slightly deviates from the data presented in (a) and (b).

Finally, the last species expected within a doped sample would be a fully exchanged Ag_2_Se particle, which would be neutral in charge, show all Ag and no Cd in EELS mapping, and present no PL emission (as these particles are not fluorescent in the visible). Figure 5 illustrates these four possible product species and how their PL and charge characteristics could combine to produce the ensemble level data as exemplified in Figures 1 and 2.

Additional EFM, EELS, and single molecule PL data was collected for a sample with 1.2 Ag/NC. These results are consistent with the conclusions reached for the other three samples already discussed. As shown in Figure S6, the CdSe NCs in this sample also consisted of many different species, with data closely resembling that of the 83 Ag/NC sample. From EFM analysis of this sample, the histogram of measured charges per NC includes a tail toward positive charges, and single molecule PL spectra exhibit either exciton or dopant PL signatures. The similarities in the optical and charge properties of the 1.2 Ag/NC and 83 Ag/NC samples, despite the drastic difference in the ICP-MS estimation of Ag/NC, further suggest that the cation exchange doping leads to heterogeneous samples with some proportion of doped NCs and some proportion of fully exchanged Ag_2_Se particles once a threshold amount of Ag^+^ is introduced.

Our findings agree with those of Routzahn et al.,^29^ who described an intermediate cation exchange state which is short-lived, is difficult to isolate, and is created at a range of different times within the full exchange process. These results also agree with the findings of Whitham et al.,^7^ who studied Cu^+^ doped CdSe NCs. In that work, either exciton PL or dopant PL was observed on the single molecule level, but never both for a single particle. Our results also help explain the differences in optical spectra for the postsynthesis versus in situ cation exchange approaches.^3,6^ For the postsynthesis cation exchange,^3,5^ a more heterogeneous sample with fewer doped NCs and more NCs with Ag near the surface producing enhanced exciton PL is likely produced. Conversely, for the one-pot cation exchange, more uniform, substitutionally doped NCs are likely being synthesized, leading to fewer undoped NCs and less enhanced exciton PL from NCs with surface Ag. This statement is also supported by the similar one-pot Cu^+^ doping experiment where more dopant PL was observed than exciton PL on the single molecule level, in a ratio of 3:1.^7^

In conclusion, CdSe NCs were doped with varying amounts of Ag^+^ cations and a number of species were observed within a single sample via EFM, EELS mapping, and single molecule PL studies. Four possible species were produced during the cation exchange Ag^+^-CdSe doping process, including undoped CdSe NCs, substitutionally Ag^+^ doped NCs, NCs with Ag near the NC surface, and fully exchanged Ag_2_Se. While the doped samples possessed a clear set of optical responses on the ensemble level, this work revealed that the ensemble is actually an average over a number of different particle types that are contributing varying amounts of distinct optical properties, and thus each particle type needs to be considered and studied on the single molecule level. This unavoidable heterogeneity in the cation exchanged doping process is important, as each particle species behaves differently. Thus, the presence of multiple species shows the difficulty of controlling the exchange process to produce a uniform sample and the importance of improving doping syntheses using cation exchange if potential applications for doped NCs are to be realized.

## Supporting Information Available

Detailed experimental methods (NC synthesis, doping, and characterization techniques), electrostatic force microscopy calculations, ICP-MS results, optical data for additional Ag^+^ doped CdSe samples, ADF-STEM images, EFM images, and data and results for an additional Ag^+^ doped CdSe sample (PDF)
