# Supplementary material for: Heterogeneity in Cation Exchange Ag+ Doping of CdSe Nanocrystals
Source: ACS Nanosci Au. 2023 Apr 25;3(4):280–5. doi: 10.1021/acsnanoscienceau.3c00010 (PMC10436366; doi:10.1021/acsnanoscienceau.3c00010)
Supplement: Supplementary file 1 — ng3c00010_si_001.pdf [file ng3c00010_si_001.pdf]

**Supporting Information**

# Heterogeneity in Cation Exchange $Ag^+$ Doping of CdSe Nanocrystals

Abigail Freyer [a], Trevor M. Tumiel [a], Michelle Smeaton [c], Benjamin H. Savitzky [d †], Lena F. Kourkoutis [e, f], Todd D. Krauss [a, b]*

[a] Department of Chemistry, University of Rochester, Rochester, New York 14627-0216, United States

[b] The Institute of Optics, University of Rochester, Rochester, New York 14627-0216, United States

[c] Department of Materials Science and Engineering, Cornell University, Ithaca, New York 14853, United States

[d] Department of Physics, Cornell University, Ithaca, New York 14853, United States

[e] School of Applied and Engineering Physics, Cornell University, Ithaca, New York 14853, United States

[f] Kavli Institute at Cornell for Nanoscale Science, Cornell University, Ithaca, New York 14853, United States

[*]Corresponding author. E-mail: krauss@chem.rochester.edu

[#]Current address: Department of Chemistry, Brown University, Providence, Rhode Island 02912, United States

## Supporting Information

## 1. Materials and Methods

The following materials and methods were also used for previously reported work.[1]

A. Chemicals and Substrates

Cadmium oxide (CdO, ≥99.99% trace metal basis), ethanol (solvent grade, 99.5%), hexanes (reagent grade, ≥98.5%), lithium nitrate ($LiNO_3$), 1-octadecene (ODE, technical grade, 90%), oleic acid (technical grade, 90%), selenium pellets (Se, <5 nm, ≥99.99% trace metal basis), silver nitrate ($AgNO_3$, 99.9999% trace metal basis), silver perchlorate ($AgClO_4$, anhydrous, 97%), toluene (solvent grade, ≥99.5%), trioctylphosphine (TOP, technical grade, 90%), and nitric acid ($HNO_3$, TraceSELECT, ≥69.0%) were purchased from Sigma-Aldrich. Calcium nitrate ($Ca(NO_3)_2$) was purchased from VWR. Polyvinyl butyral (PVB, Butvar B-98) was purchased from Fisher Scientific. Cadmium (Cd, 10 ppm in $HNO_3$), indium (In, 10 ppm in $HNO_3$), selenium (Se, 10 ppm in $HNO_3$), and silver (Ag, 10 ppm in $HNO_3$) ICPMS standards were purchased from Inorganic Ventures. All chemicals were used as delivered without further purification. Highly oriented pyrolytic graphite (HOPG, grade SPI-2) was purchased from SPI Supplies.

B. Synthesis of CdSe Nanocrystals

CdSe NCs were made with known synthesis procedures.[2,3] A 0.2 M cadmium oleate precursor was produced by adding CdO (3.18 g, 24.8 mmol), ODE (90 mL, 280 mmol), and oleic acid (34.5 mL, 108 mmol) together in a 250-mL round bottom flask. With a condenser, stirring, and under $N_2$ flow, the reaction flask was heated to 220°C for about one hour until all components were dissolved. The flask was cooled until the solution became a white, waxy solid. A 1 M trioctylphosphine selenide (TOPSe) precursor was produced by dissolving Se pellets (0.7896 g, 10 mmol) in TOP (10 mL, 22 mmol) in a $N_2$-filled glovebox. The reaction mixture was heated to 60°C with stirring until dissolved.

For the NC synthesis, the cadmium oleate precursor was melted at approximately 60°C under $N_2$ with stirring. In a 100-mL flask, 0.2 M cadmium oleate (30 mL, 6 mmol) and ODE (20 mL, 62 mmol) were heated to 270°C under $N_2$ with stirring. Once at 270°C, 1 M TOPSe (2 mL, 2 mmol) was injected rapidly. Immediately following TOPSe injection, the reaction temperature was set to 220°C and the NCs were allowed to grow for 6 minutes. After 6 minutes, the NC solution was cooled to room temperature. This synthesis consistently produced CdSe NCs with an average diameter of 2.9-3.2 nm.

To ensure the removal of excess ligands and to redisperse in an alternate solvent, a rigorous washing procedure was followed. The NCs were washed by precipitation in ethanol with centrifugation. The NCs were redispersed in hexanes and the precipitation step was repeated two additional times with final redispersion of the NCs in toluene.

C. Doping of CdSe Nanocrystals with Cations

The CdSe NCs were doped with $Ag^+$ ions following the cation exchange doping procedure established by Sahu *et al.*[4] Samples with a range of added $Ag^+$ were prepared in order to study the effects of dopant concentration. First, the concentration of the washed NC solution was determined from the absorbance following the method described by Yu *et al.*[5] From this concentration and the

assumed composition of the CdSe unit cell, the Cd concentration was calculated. $Ag^+$ was then added in different Ag:Cd ratios based on this calculated Cd concentration to create the range of dopant concentrations. $AgNO_3$ was used as the Ag precursor; 0.1 M and 0.02 M $AgNO_3$ solutions in ethanol were prepared and used for doping. The different $AgNO_3$ concentrations were used in order to keep the added volume of the ethanolic solution approximately constant and to maintain a similar end volume.

For the doping procedure, an oil bath was heated to 60°C on a stir plate. Vials with the NC toluene solution were placed in the oil bath with stirring. After the temperature of the NC solution equilibrated (roughly 5 minutes), TOP was added at a volume of 5% of the initial NC solution volume. Precisely 30 seconds later, the $AgNO_3$ in ethanol was added (volume and concentration depending on the desired Ag:Cd ratio). One minute later, ethanol was added (2.5x the original NC solution volume) in order to quench the cation exchange doping reaction. After 3 minutes, the solution was centrifuged to allow for NC precipitation. The resulting NCs were dispersed in toluene (volume equal to the original NC solution volume). Typically, this doping procedure was carried out on a NC solution volume of 3-4 mL.

D. Sample Characterization

Ultraviolet-visible (UV-Vis) absorption spectrophotometry, fluorescence spectroscopy, inductively coupled plasma-mass spectrometry (ICP-MS), atomic and electrostatic force microscopies (AFM and EFM), high angle annular dark field-transmission electron microscopy (HAADF-TEM) electron energy loss spectroscopy (EELS), and single molecule PL microscopy were used to characterize the size, composition, and optical and electrostatic properties of the doped NCs.

Ensemble optical characterization was performed on NCs dispersed and diluted in toluene in a 1 cm path length quartz cuvette. Absorption spectra were obtained using a PerkinElmer Lambda 950 UV/VIS spectrophotometer. Photoluminescence (PL) spectra were collected using a home-built fluorometer system with a 450 W xenon arc lamp source coupled to an excitation SpectraPro 150 monochromator. A photomultiplier tube (PMT) was used for PL detection with an emission SpectraPro 300i monochromator every 1 nm with an integration time of 100 ms. The excitation wavelength was 480 nm for all measured PL spectra.

ICP-MS analysis was performed with an Agilent 7900 ICP-MS system. For calibration, the sample data was compared to intensities of four separate single-element standards: Ag, Cd, Se, and In (purchased from Inorganic Ventures). In was used as an internal standard. For ICP-MS, the samples were prepared first by precipitating out the NCs by adding ethanol and centrifuging. The NCs were allowed to dry and then the NC pellet was digested in spectroscopy-grade concentrated nitric acid. The NC nitric acid solution was diluted, resulting in a ~2% nitric acid solution with <10 ppm concentrations of Cd, Se, and Ag. The Ag:Cd ratio was determined using the ratio of the respective concentrations for each solution and this ratio was used to calculate the Ag/NC concentration based off the theoretical Cd/NC.

E. Electrostatic Force Microscopy Measurements

*EFM sample preparation*: A thin layer of PVB was coated onto the surface of the HOPG substrate by spin coating 30 μL of a 0.05% PVB in toluene solution for 60 seconds at 3000-4000 rpm. A dilute QD solution (in toluene) was then spin coated, again 30 μL for 60 seconds at 3000-4000 rpm.

AFM and EFM images were obtained at room temperature with an Asylum MFP-3D-BIO AFM inside an acoustic hood purged with $N_2$ to <17% relative humidity using the Asylum Research Version 12 software. Olympus-made AC240TM-R3 titanium-platinum-coated silicon cantilevers with spring constants of ~1.2-1.8 N/m from Asylum Research were used at their resonant frequencies of 62-71 kHz. In order to obtain topographical AFM images and electrostatic EFM images simultaneously, the microscope made two passes for each scan. Two lock-in amplifiers were used to simultaneously measure the $\Delta\nu(\omega)$ and $\Delta\nu(2\omega)$ signals. Typical parameters were: $V_{ac}$ = 3 V peak-to-peak, $\omega$ = 400 Hz, lock-in time constant = 3 ms, scan rate = 0.75 Hz per line, and lift-height $z$ = 5-8 nm. The acquisition time for a complete image was approximately 11 minutes. Images were recorded such that $V_{dc} = -\phi$ (typically, $|V_{dc}| < 0.7$ V). Curve fitting and image analysis were performed with Igor Pro 6.3.7.2 and calculations were performed with Wolfram *Mathematica* 10.

F. HAADF-STEM EELS

In order to clean up the sample for HAADF-TEM imaging, the NC solutions were washed by precipitation with ethanol and redispersion in hexanes an additional three times. The final NC solutions used for spotting were in hexanes. Samples were drop cast onto TEM grids with an ultrathin, nominally 2-3 nm amorphous carbon support layer. Imaging was performed on an aberration-correct FEI Titan operating in HAADF-STEM mode at an accelerating voltage of 60 keV and with a 21 mrad convergence semi-angle. To minimize sample contamination, data was acquired under in-situ liquid nitrogen cooling. To account for stage instabilities introduced by cryogenic cooling and obtain high SNR atomic resolution images, fast acquisition image stacks were obtained, aligned, and averaged.[6]

G. Single Molecule PL Microscopy

Single molecule PL measurements were obtained using a home-built scanning confocal microscopy set-up. Approximately 50 µL of a 1 wt% PMMA in toluene solution was spin coated onto a quartz coverslip, followed by 15 µL of diluted NC solution. An additional 15 µL of 1 wt% PMMA was added to cover the NCs to avoid oxidation. Samples were excited at 488 nm using a Melles Griot 43 Series Ion Laser and emission was collected using a CCD detector (Princeton Instruments). Excitation power was held at roughly 0.5 $kW/cm^2$ (varied slightly for samples with different Ag dopant levels to maximize results) and a 40x ELWD objective (Nikon) was used. A 488 nm long pass filter was used in the emission pathway. Integration time for spectra acquisition was held at 30 s.

H. Safety Considerations

Before utilizing the chemicals included in the sections above, all safety data sheets should be reviewed to ensure proper handling. In particular, CdO is a known carcinogen and is fatal if inhaled. In addition to exclusively handling CdO in a well-ventilated area, respiratory protection should be added to the standard laboratory personal protective equipment (PPE). All volatile solvents should be handled with extreme care to avoid fires. TOP, a pyrophoric substance, should be accessed in a glove box to avoid exposure to $O_2(g)$.

When operating a laser, as is required for the single molecule PL measurements discussed above, proper safety precautions must be taken to avoid injury. In particular, laser safety goggles (graded for the wavelength range being accessed) should be worn when operating the laser at all times. Low laser output powers should be used whenever possible to further reduce the dangers of scattered laser light.

## 2. Electrostatic Force Microscopy Explanation

A. Equations and theory

EFM measures electrostatic forces between a conductive cantilever and conductive substrate in a modified AFM experiment. EFM consists of two passes of a line scan, a first pass equal to a normal AFM pass and a second pass with the tip lifted off the surface and scanned with an applied voltage.[7,8] The attractive force between the cantilever and the substrate with the applied voltage is proportional to the square of the voltage difference between the cantilever and the substrate.

$$F = \frac{1}{2}\frac{\partial C}{\partial z}V^2 \qquad \text{(S1)}$$

The application of a sinusoidal voltage, $V=V_{dc}+V_{ac}sin(wt)$, results in an electrostatic attraction with components at zero frequency, at the frequency of the applied voltage, $\omega$, and at twice that frequency, $2\omega$. With lock-in amplification, the components of the force on the tip at $\omega$ and $2\omega$, the capacitive and Coulombic forces, respectively, can be determined:[9,10]

$$F(\omega) = \left(\frac{\partial C}{\partial z}(V_{dc} + \phi) + \frac{QC}{4\pi\epsilon_0(z+R)^2} + \frac{Q_1 C}{4\pi\epsilon_0\left(z+R+\frac{2h}{\epsilon_1}\right)^2} + \frac{\partial C}{\partial z}\frac{Q_2}{C}\right)V_{ac} \qquad \text{(S2)}$$

$$F(2\omega) = \frac{\partial C}{\partial z}\frac{{V_{ac}}^2}{4}. \qquad \text{(S3)}$$

The EFM tip is modeled as a cone with a sphere end with radius $R$. $C$ is the capacitance between the EFM tip and the substrate, $z$ is the separation between the insulator surface and the bottom of the EFM tip, and $\phi$ is the contact potential difference between the tip and the substrate. The samples consist of a metallic highly oriented pyrolitic graphite substrate with the nanocrystals atop a thin insulator layer with thickness $h$ and dielectric constant $\varepsilon_1$. $Q_1$ and $Q_2$ are induced charges on the metallic substrate and the EFM tip, and assuming a parallel plate geometry between the tip and substrate,

$$Q_1 = -Q\frac{z}{\left(\frac{h}{\epsilon}+z\right)} \qquad \text{(S4)}$$

$$Q_2 = -Q\left(1 - \frac{z}{\left(\frac{h}{\epsilon}+z\right)}\right). \qquad \text{(S5)}$$

The capacitance of the tip-substrate system can be measured and subsequently used to determine the surface charge, $Q$, from the measured force gradient on the tip at $\omega$.[11] Dielectric properties can be determined by fitting the measured force on the cantilever at $2\omega$.[10] EFM allows for the facile determination of the dielectric constants and surface charges of individual NCs. The response of the oscillating cantilever at twice the frequency of the applied voltage, $\Delta v(2\omega)$, yields the capacitive force information from which the dielectric constant can be determined. An increase in magnitude in the presence of a NC is expected due to the larger dielectric constant of CdSe compared to the

surroundings. Similarly, the response of the cantilever at the frequency of the applied voltage, $\Delta v(\omega)$, measures local electrostatic potential variations and allows for determination of the charge magnitude. For a charge image, with $V_{dc}$ set to zero out the contact potential difference, three types of behavior are possible: an increase or decrease in the measured Coulombic force corresponding to a positive or negative charge, respectively, or a static force corresponding to a neutral NC.

## 3. Additional Data

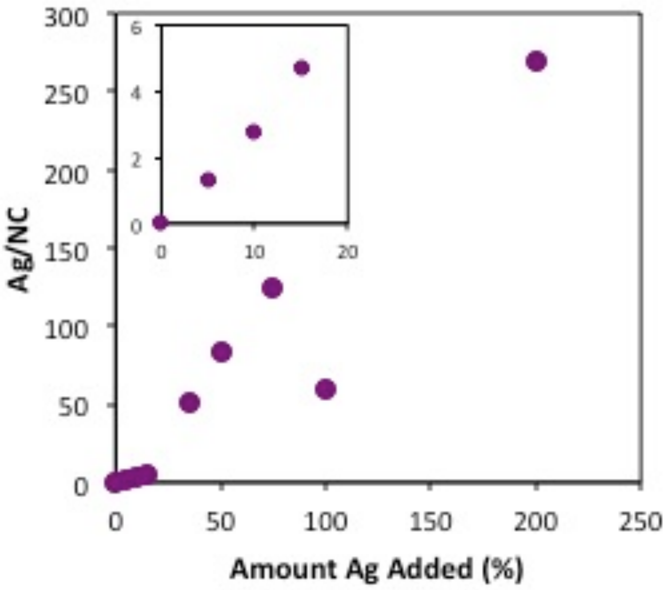


**Figure S1.** The number of incorporated Ag atoms per NC, determined by ICP-MS vs. the amount of Ag added to the exchange reaction, reported as a Ag/Cd molar percentage. As expected, as the amount of Ag added increases, the number of Ag/NC also increases.

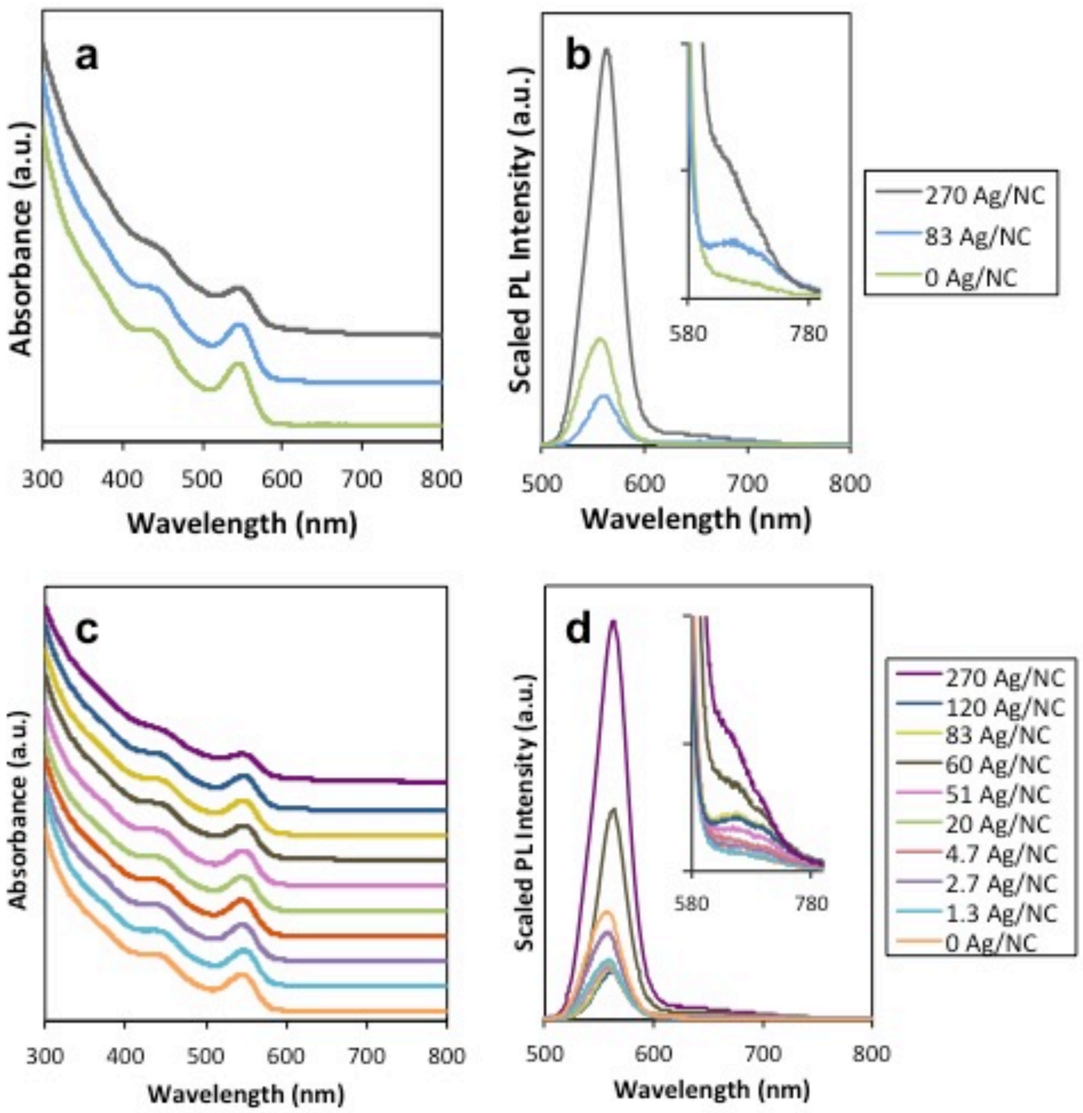


**Figure S2.** Ensemble (a) absorbance and (b) PL spectra for two $Ag^+$ doped CdSe samples and the corresponding undoped CdSe sample from a single series of doping. Ensemble (c) absorbance and (d) PL spectra for the full series of $Ag^+$ doped CdSe samples and the corresponding undoped CdSe sample from which the samples described in (a) and (b) were taken. The insets in (b) and (d) magnify the weak fluorescence feature near 700 nm.

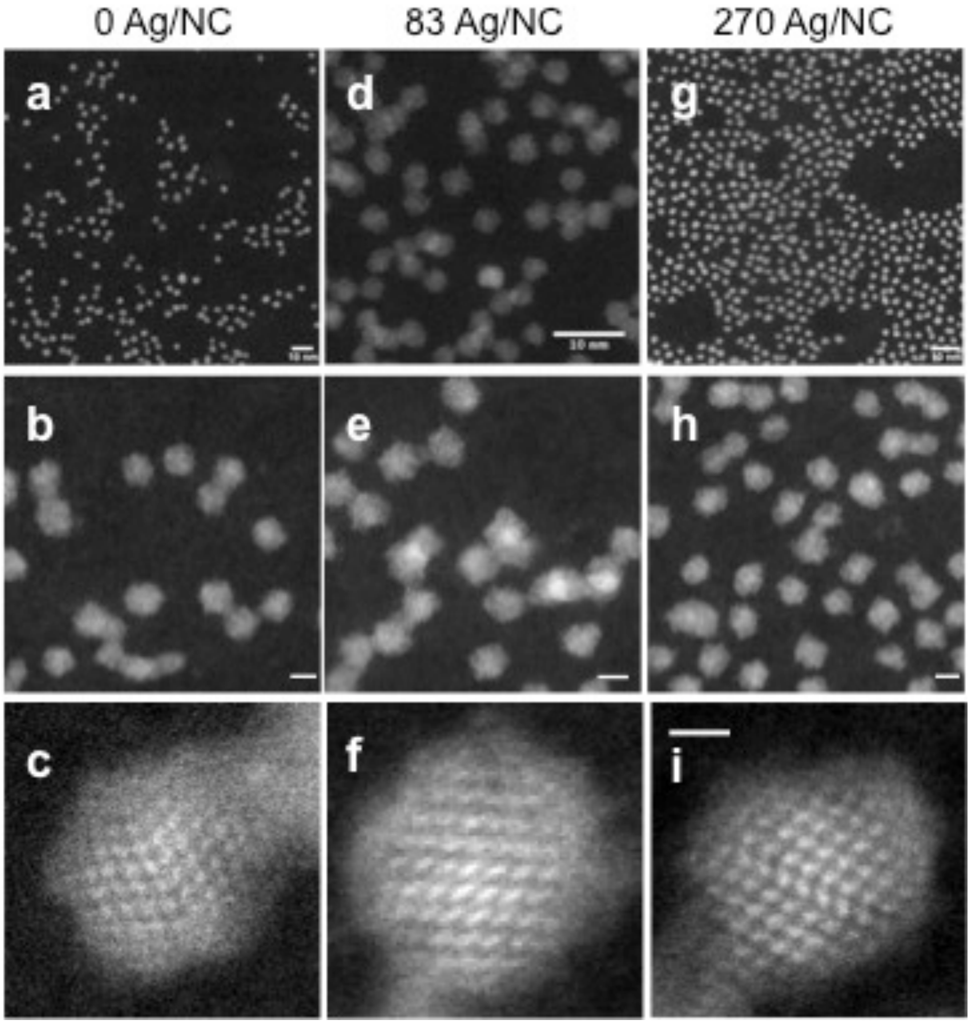


**Figure S3.** ADF-STEM images for samples of (a,b,c) 0 Ag/NC, (d,e,f) 83 Ag/NC, (g,h,i) 270 Ag/NC with increasing magnification going down the columns. Scale bars in a,d,g represent 10 nm, in b,e,f represent 3 nm, and in bottom images represents 1 nm.

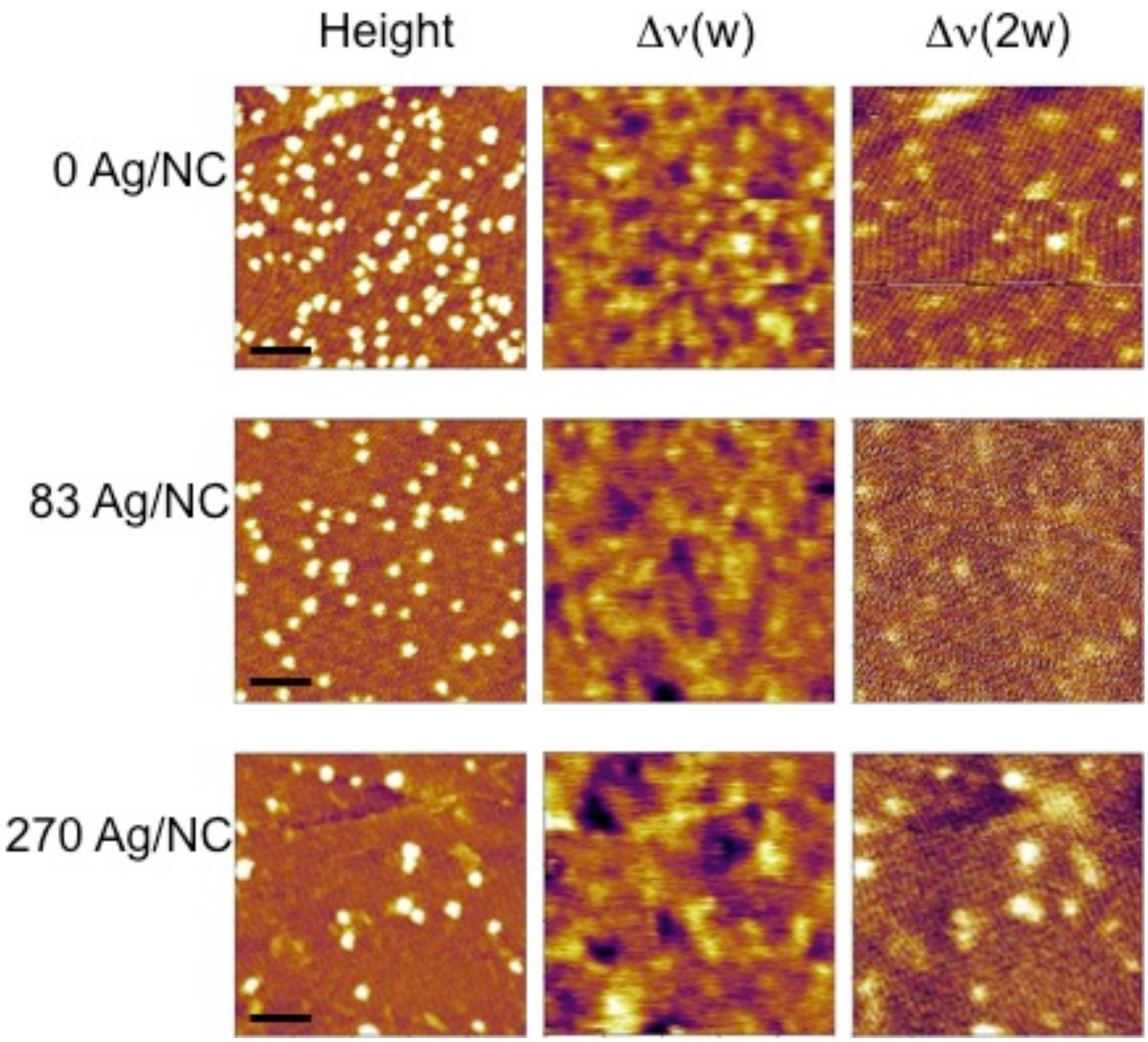


**Figure S4.** EFM images for three samples of $Ag^{+}$ doped CdSe NCs with the height, charge, and dielectric images given left to right for each sample. Scale bars represent 100 nm.

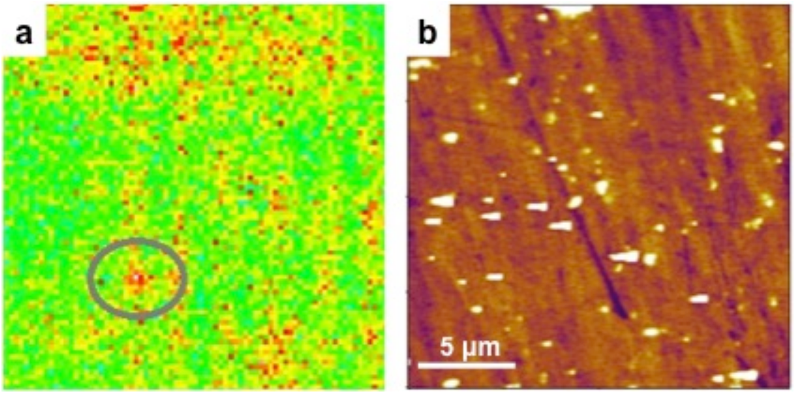


**Figure S5.** Representative (a) single molecule PL map and (b) AFM height image of a correlated area on a spun out $Ag^{+}$:CdSe sample with 270 Ag/NC. The two images represent similar imaging areas. The circle in (a) highlights the only NC observed in this single molecule PL map, which was confirmed by collecting a PL spectral map of the same region of interest. On average, a single NC was observed within this imaging area with PL while about 70 particles were seen within the same area size in AFM.

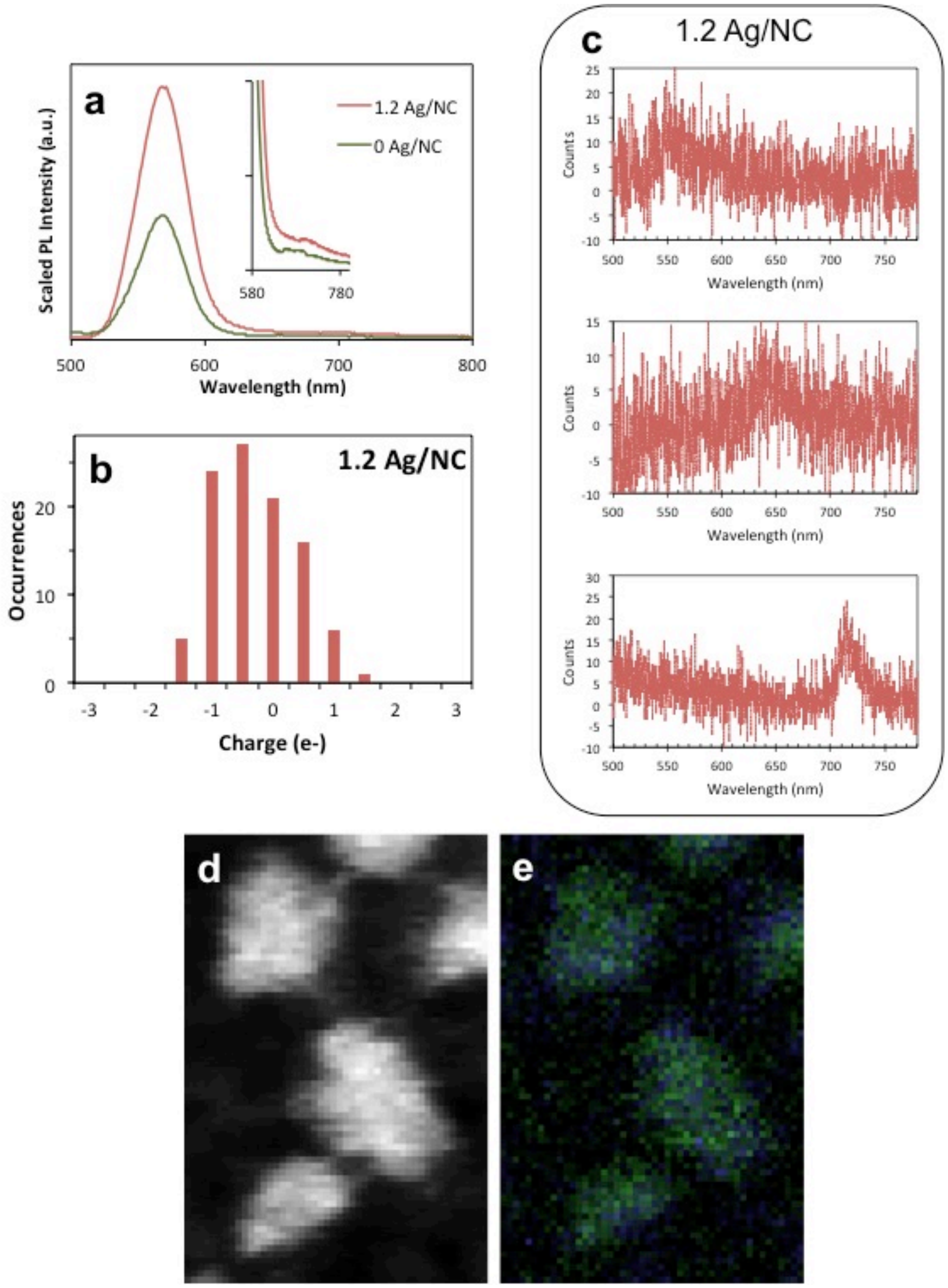


**Figure S6.** (a) Ensemble level PL spectra for a 1.2 Ag/NC sample and its corresponding undoped sample (0 Ag/NC). (b) Charge histogram for a 1.2 Ag/NC sample. (c) Sample single molecule PL spectra for the sample of 1.2 Ag/NC showing both the exciton PL and the dopant PL within the sample. (d) Annular dark field (ADF) image and (e) corresponding overlaid Ag (blue) and Cd (green) EELS maps for the 1.2 Ag/NC $Ag^+$ doped CdSe sample.

## 4. References

1. Freyer, A. R.; Sercel, P. C.; Hou, Z.; Savitzky, B.; Kourkoutis, L. F.; Efros, A. L.; Krauss, T. D., Explaining the Unusual Photoluminescence of Semiconductor Nanocrystals Doped via Cation Exchange. *Nano Lett.* **2019,** *19*, 4797-4803.

2. Yu, W. W.; Peng, X., Formation of High-Quality CdS and Other II–VI Semiconductor Nanocrystals in Noncoordinating Solvents: Tunable Reactivity of Monomers. *Angew. Chem., Int. Ed.* **2002,** *41* (13), 2368-2371.

3. Bullen, C. R.; Mulvaney, P., Nucleation and Growth Kinetics of CdSe Nanocrystals in Octadecene. *Nano Lett.* **2004,** *4* (12), 2303-2307.

4. Sahu, A.; Kang, M. S.; Kompch, A.; Notthoff, C.; Wills, A. W.; Deng, D.; Winterer, M.; Frisbie, C. D.; Norris, D. J., Electronic Impurity Doping in CdSe Nanocrystals. *Nano Lett.* **2012,** *12* (5), 2587-2594.

5. Yu, W. W.; Qu, L.; Guo, W.; Peng, X., Experimental Determination of the Extinction Coefficient of CdTe, CdSe, and CdS Nanocrystals. *Chem. Mater.* **2003,** *15* (14), 2854-2860.

6. Savitzky, B. H.; El Baggari, I.; Clement, C. B.; Waite, E.; Goodge, B. H.; Baek, D. J.; Sheckelton, J. P.; Pasco, C.; Nair, H.; Schreiber, N. J.; Hoffman, J.; Admasu, A. S.; Kim, J.; Cheong, S.-W.; Bhattacharya, A.; Schlom, D. G.; McQueen, T. M.; Hovden, R.; Kourkoutis, L. F., Image registration of low signal-to-noise cryo-STEM data. *Ultramicroscopy* **2018,** *191*, 56-65.

7. Krauss, T. D.; Brus, L. E., Charge, Polarizability, and Photoionization of Single Semiconductor Nanocrystals. *Phys. Rev. Lett.* **1999,** *83* (23), 4840-4843.

8. Cherniavskaya, O.; Chen, L.; Weng, V.; Yuditsky, L.; Brus, L. E., Quantitative Noncontact Electrostatic Force Imaging of Nanocrystal Polarizability. *J. Phys. Chem. B* **2003,** *107* (7), 1525-1531.

9. Krauss, T. D.; Brus, L. E., Electronic properties of single semiconductor nanocrystals: optical and electrostatic force microscopy measurements. *Mater. Sci. Eng.* **2000,** *B69-70*, 289-294.

10. Krauss, T. D.; O'Brien, S.; Brus, L. E., Charge and Photoionization Properties of Single Semiconductor Nanocrystals. *J. Phys. Chem. B* **2001,** *105* (9), 1725-1733.

11. Cherniavskaya, O.; Chen, L.; Islam, M. A.; Brus, L., Photoionization of Individual CdSe/CdS Core/Shell Nanocrystals on Silicon with 2-nm Oxide Depends on Surface Band Bending. *Nano Lett.* **2003,** *3* (4), 497-501.
